# Supplementary figures and images for: Family-based genome-wide association study of leprosy in Vietnam
Source: PLoS Pathog. 2020 May 18;16(5):e1008565. doi: 10.1371/journal.ppat.1008565 (PMC7259797; doi:10.1371/journal.ppat.1008565)

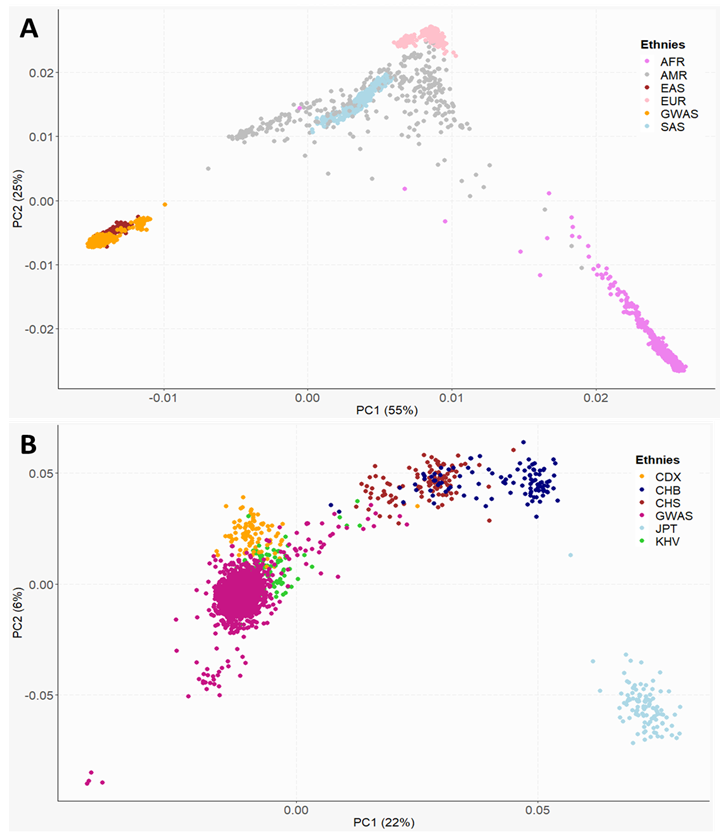

Supplement: S1 Fig — Principal component analysis of the GWAS discovery sample with all 1000 genomes phase 3 samples (A) and with the East-Asian 1000 genomes samples (B) AFR = African; AMR = American; EAS = East Asian; EUR = European; GWAS = Genome-wide association study samples; SAS = South Asian; CDX = Chinese Dai in Xishuangbanna, China; CHB = Han Chinese in Beijing, China; CHS = Southern Han Chinese; JPT = Japanese in Tokyo, Japan; KHV = Kinh in Ho Chi Minh City, Vietnam. (TIF) [file ppat.1008565.s012.tif]

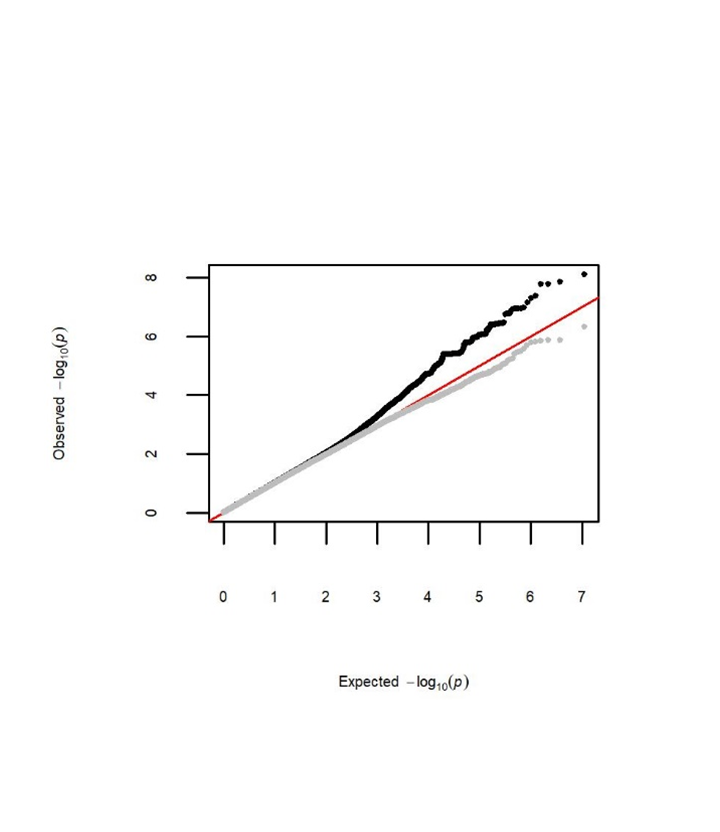

Supplement: S2 Fig — (TIF) [file ppat.1008565.s013.tif]
